# Effect of stem cell therapies on tendon-bone healing after anterior cruciate ligament reconstruction in animal models: a systematic review and meta-analysis

**DOI:** 10.3389/fbioe.2026.1869863

**Published:** 2026-10-20

**Authors:** Xuedong Sun, Shibo Zhao, Congcong Wang

**Affiliations:** Department of Joint Surgery, Weifang People’s Hospital, Shandong Second Medical University Weifang, Weifang, Shandong, China

**Keywords:** ACL reconstruction, animal models, mesenchymal stem cells, meta-analysis, tendon–bone healing

## Abstract

**Background:**

Poor tendon-bone healing is a major contributor to graft failure after anterior cruciate ligament (ACL) reconstruction. Stem cell-based approaches, including mesenchymal stem cells (MSCs) and their derivatives, have shown promise in animal models, but findings remain heterogeneous.

**Methods:**

PubMed, Embase, Scopus, SPORTDiscus, and the Cochrane Library were systematically searched from inception to December 2025 for controlled animal studies evaluating stem cell-based interventions in ACL reconstruction. Outcomes included biomechanical properties (ultimate failure load and stiffness), bone volume fraction (BV/TV) assessed by micro-CT, and histological integration. Risk of bias was assessed using SYRCLE’s tool. Pooled standardized mean differences (SMDs) were calculated using random-effects models.

**Results:**

Sixteen controlled animal studies were included, involving diverse stem cell types, including bone marrow-derived mesenchymal stem cells (BMSCs), adipose-derived stem cells (ADSCs), tendon-derived stem cells (TDSCs), and synovium-derived mesenchymal stem cells (SMSCs), as well as different delivery strategies. Compared with controls, stem cell-based interventions significantly improved tendon-bone healing outcomes, including ultimate failure load (SMD = 1.34, 95% CI 0.93–1.75), stiffness (SMD = 0.96, 95% CI 0.64–1.27), BV/TV (SMD = 1.03, 95% CI 0.75–1.31), and histological integration (SMD = 0.86, 95% CI 0.59–1.13), all P < 0.00001. Substantial heterogeneity was observed across outcomes, and the overall risk of bias was moderate, reflecting variability in animal species, cell sources, delivery methods, and follow-up durations.

**Conclusion:**

In animal models of ACL reconstruction, stem cell-based therapies significantly improve biomechanical properties, including ultimate failure load and stiffness, as well as bone formation and histological integration. The most pronounced effects were observed during the early healing phase, particularly at 4–6 weeks. Standardized preclinical protocols and longer-term evaluations are needed before clinical translation.

## Introduction

Anterior cruciate ligament (ACL) rupture is a clinically important knee ligament injury, particularly affecting young and physically active individuals. It is estimated that over two million ACL injuries occur annually worldwide, with incidence continuing to rise as participation in high-impact sports increases (van Melick et al., 2025). Surgical reconstruction remains a standard treatment for complete ACL tears, aiming to restore knee stability, facilitate return to pre-injury activity levels, and reduce secondary meniscal or cartilage damage, although its role in preventing long-term knee osteoarthritis remains controversial (Rothrauff et al., 2020). Despite advances in surgical techniques and rehabilitation protocols, graft failure rates remain as high as 10%–20%, with insufficient tendon-bone healing being a leading cause of reconstruction failure (Mayer et al., 2025). Inadequate integration at the graft-bone interface compromises mechanical stability, predisposes to tunnel widening, and ultimately leads to poor functional outcomes.

To address this critical limitation, regenerative medicine approaches have been actively explored. Among these, stem cell-based therapies have emerged as one of the most promising strategies. Mesenchymal stem cells (MSCs), extracted from diverse tissue sources such as bone marrow, adipose tissue, synovium, tendon, and umbilical cord blood, possess multipotent differentiation potential, immunomodulatory capacity, and paracrine activity through the secretion of trophic factors (Huang et al., 2020). These properties enable MSCs to modulate inflammation, promote revascularization, and facilitate fibrocartilage and new bone formation at the tendon-bone interface (Zhang et al., 2024). Beyond direct cell transplantation, stem cell-derived extracellular vesicles (EVs) and exosomes have attracted attention for their ability to recapitulate many therapeutic effects of MSCs while avoiding risks such as ectopic differentiation or immune rejection (Yang et al., 2023; Song et al., 2025). Additionally, the integration of stem cells into biomaterial scaffolds and three-dimensional (3D) bio-printed constructs has further advanced tissue engineering approaches to augment tendon-bone healing (Wang et al., 2023; Wang et al., 2024).

A growing number of preclinical animal studies have investigated the use of stem cell therapies to improve tendon-bone healing after ACL reconstruction. Positive findings have been reported, such as enhanced biomechanical properties, increased bone volume fraction within graft tunnels, and improved histological maturation of the graft-bone interface. For instance, bone marrow-derived MSCs have been shown to accelerate mineralized fibrocartilage formation, while adipose-derived stem cells promoted collagen fiber alignment and early vascularization (Yanuar et al., 2025). Tendon-derived stem cells and synovium-derived MSCs have also been tested, demonstrating potential benefits in graft remodeling (Yuan et al., 2024). Furthermore, bioengineered strategies, such as mesenchymal stem cell-laden three-dimensional bio-printed scaffold sleeves and bioactive decellularized tendon-derived stem cell sheets, have been developed to improve local biological support and enhance graft healing at the tendon-bone interface (Yao et al., 2023; Park et al., 2018).

Nevertheless, the evidence remains heterogeneous, and the reported effects of stem cell-based interventions vary across outcomes and follow-up durations. For example, Kosaka et al. reported that differences in ultimate failure load and stiffness were no longer statistically significant at 8 and 12 weeks, despite early improvements. Matsumoto et al. similarly observed significant improvements in ultimate failure load at 2 and 4 weeks, but not at 8, 16, or 24 weeks. In addition, Soon et al. reported higher load-to-failure in MSC-enhanced grafts, whereas stiffness and Young’s modulus were lower than those in controls (Kosaka et al., 2016; Matsumoto et al., 2021; Soon et al., 2007). These findings indicate that the efficacy of stem cell-based interventions may be time-dependent and outcome-specific, and the current evidence does not allow definitive conclusions regarding the reasons for inconsistent or non-significant results. The small sample sizes and variable methodological quality of many animal experiments further complicate interpretation. Moreover, outcomes across studies are diverse, encompassing biomechanical, imaging, and histological measures, making direct comparison difficult. To date, no comprehensive synthesis has systematically evaluated the magnitude and consistency of treatment effects across animal studies.

Systematic reviews and meta-analyses of preclinical evidence are increasingly recognized as essential tools for informing translational research. Previous reviews on related ligament, tendon, and tendon-bone healing fields have suggested that stem cell-based or biologic augmentation may improve early histological, biomechanical, imaging, or structural healing outcomes, particularly in preclinical models. For example, systematic reviews of adult stem cells in ACL treatment and tendon graft ligamentization after ACL reconstruction have reported potential benefits in early graft healing, while also emphasizing the small number of available studies, heterogeneity of experimental protocols, and uncertainty regarding the optimal cell source and delivery strategy (Guo et al., 2018; Wang et al., 2022). In broader tendon disorders, meta-analytic evidence from prospective clinical studies has also suggested possible improvements in pain, function, and imaging outcomes after MSC therapy, but the overall evidence remains limited and requires confirmation in larger controlled studies (Cho et al., 2021). Therefore, a focused quantitative synthesis of animal studies on tendon-bone healing after ACL reconstruction is particularly valuable, as it can provide pooled effect estimates, identify sources of inconsistency, and clarify methodological limitations that need to be addressed before clinical translation.

Therefore, the present study aims to conduct the first systematic review and meta-analysis of controlled animal experiments evaluating stem cell-based therapies for tendon-bone healing after ACL reconstruction. By integrating biomechanical, micro-computed tomography (micro-CT), and histological outcomes across diverse preclinical models, we seek to 1. determine whether stem cell interventions consistently enhance graft-bone integration; 2. explore potential moderators of treatment effect, such as cell source, delivery method, animal species, and follow-up duration; and 3. assess the overall methodological quality of the evidence. The findings are expected to provide a robust evidence base to guide the design of future preclinical studies and inform the early-phase clinical translation of stem cell-assisted ACL reconstruction.

## Methods

### Protocol registration

This systematic review and meta-analysis was conducted in accordance with the *Preferred Reporting Items for Systematic Reviews and Meta-Analyses* (PRISMA) 2020 guidelines. The review protocol was developed following the PRISMA statement and was prospectively registered in the PROSPERO (CRD420251137985).

### Eligibility criteria

Studies were considered eligible if they met the following inclusion criteria:

Study design: controlled *in vivo* animal experiments.

Population: animal models undergoing ACL reconstruction.

Intervention: stem cell-based therapies, including mesenchymal stem cells (MSCs) derived from bone marrow, adipose tissue, tendon, synovium, or umbilical cord blood, as well as stem cell-derived products such as extracellular vesicles, exosomes, or scaffold-based delivery systems.

Comparator: animals undergoing the same ACL reconstruction procedure as the intervention group but without stem cell treatment, including untreated ACL reconstruction controls, vehicle controls, scaffold-only controls, or other non-cellular controls. Sham-operated or non-reconstructed animals were not used as control groups for the quantitative meta-analysis.

Outcomes: at least one relevant measure of tendon-bone healing, including a. biomechanical properties (e.g., ultimate failure load, stiffness); b. micro-CT evaluation of bone volume fraction (BV/TV) or mineralization; or c. histological assessment of graft-bone integration.

No language restriction was applied during study screening. The studies ultimately included in this review were published in English or Chinese, and Chinese-language articles were assessed by reviewers fluent in Chinese.

Exclusion criteria were: 1. clinical or human studies, case reports, reviews, editorials, or letters; 2. *in vitro* studies without animal models; 3. studies without a control group; 4. studies not reporting tendon-bone healing outcomes; 5. duplicate publications; and 6. conference abstracts with insufficient data or studies for which full text could not be retrieved.

### Information sources and search strategy

A comprehensive search of PubMed, Embase, Scopus, SPORTDiscus, and the Cochrane Library was conducted from database inception to December 2025. The search strategy combined keywords and MeSH terms related to *“anterior cruciate ligament reconstruction”*, *“stem cells”*, and *“animal models”*. The complete search strings for each database are provided in Supplementary Appendix S1. In addition, reference lists of all included studies and relevant reviews were manually screened to identify additional eligible studies.

### Study selection

All retrieved references were imported into EndNote V.21 (Clarivate Analytics) for deduplication. Two reviewers (SZ and CW) independently screened titles and abstracts to identify potentially eligible studies, followed by full-text assessment against the inclusion and exclusion criteria. Discrepancies were resolved by discussion or consultation with a third reviewer. Reasons for exclusion at the full-text stage were documented. The study selection process was summarized using a PRISMA 2020 flow diagram.

### Data extraction

Two reviewers (SZ and CW) independently extracted data using a standardized form. Extracted information included:

bibliographic details (first author, year, country);
animal model (species, sample size, follow-up duration);
type and source of stem cells;
delivery method (injection, scaffold, graft wrapping, bio-printing);
control intervention;
outcome measures (biomechanical, micro-CT, histological);
risk of bias assessment.

Any disagreements were resolved through discussion. If necessary, study authors were contacted for clarification.

### Risk of bias assessment

Methodological quality of included studies was evaluated independently by two reviewers using the SYRCLE’s risk of bias tool for animal experiments. This tool assesses ten domains: sequence generation, baseline characteristics, allocation concealment, random housing, blinding of caregivers, random outcome assessment, blinding of outcome assessors, incomplete outcome data, selective outcome reporting, and other biases. Each domain was judged as “low,” (L) “unclear,” (U) or “high”(H) risk. Discrepancies were resolved by consensus or third-party adjudication.

### Data synthesis and statistical analysis

Data were analyzed using Review Manager. Continuous outcomes were expressed as mean differences (MDs) with 95% confidence intervals (CIs). When different scales were used, standardized mean differences (SMDs) were calculated. A random-effects model was applied to account for inter-study heterogeneity. Statistical heterogeneity was assessed using the χ^2^ test (p < 0.10 indicating significance) and quantified with the I^2^ statistic, with thresholds of 25%, 50%, and 75% representing low, moderate, and high heterogeneity, respectively. Planned subgroup analyses included stratification by follow-up duration.

### Ethics and dissemination

As this review was based exclusively on published animal data, ethical approval was not required. Findings will be disseminated through publication in a peer-reviewed journal and presentations at scientific conferences.

## Results

### Study selection

The database search yielded a total of 4,528 records. After removal of 2,130 duplicates, 2,398 records remained for title and abstract screening. Of these, 2,236 records were excluded as irrelevant. The full texts of 162 potentially eligible reports were sought for retrieval; 30 reports could not be obtained. Therefore, 132 full-text articles were assessed for eligibility. Among these, 116 studies were excluded for the following reasons: no control group, no relevant tendon-bone healing outcomes, not original research, case reports, editorials or letters, or reviews. Ultimately, 16 studies met the inclusion criteria and were included in the systematic review and meta-analysis (Figure 1, PRISMA flow diagram). All quantitative comparisons were conducted within ACL reconstruction models. In each included comparison, the experimental group underwent ACL reconstruction combined with a stem cell-based intervention, whereas the control group underwent ACL reconstruction without stem cell treatment or with a corresponding vehicle/scaffold-only control. No sham-operated or non-reconstructed animals were included as control groups in the meta-analysis.

**FIGURE 1:**
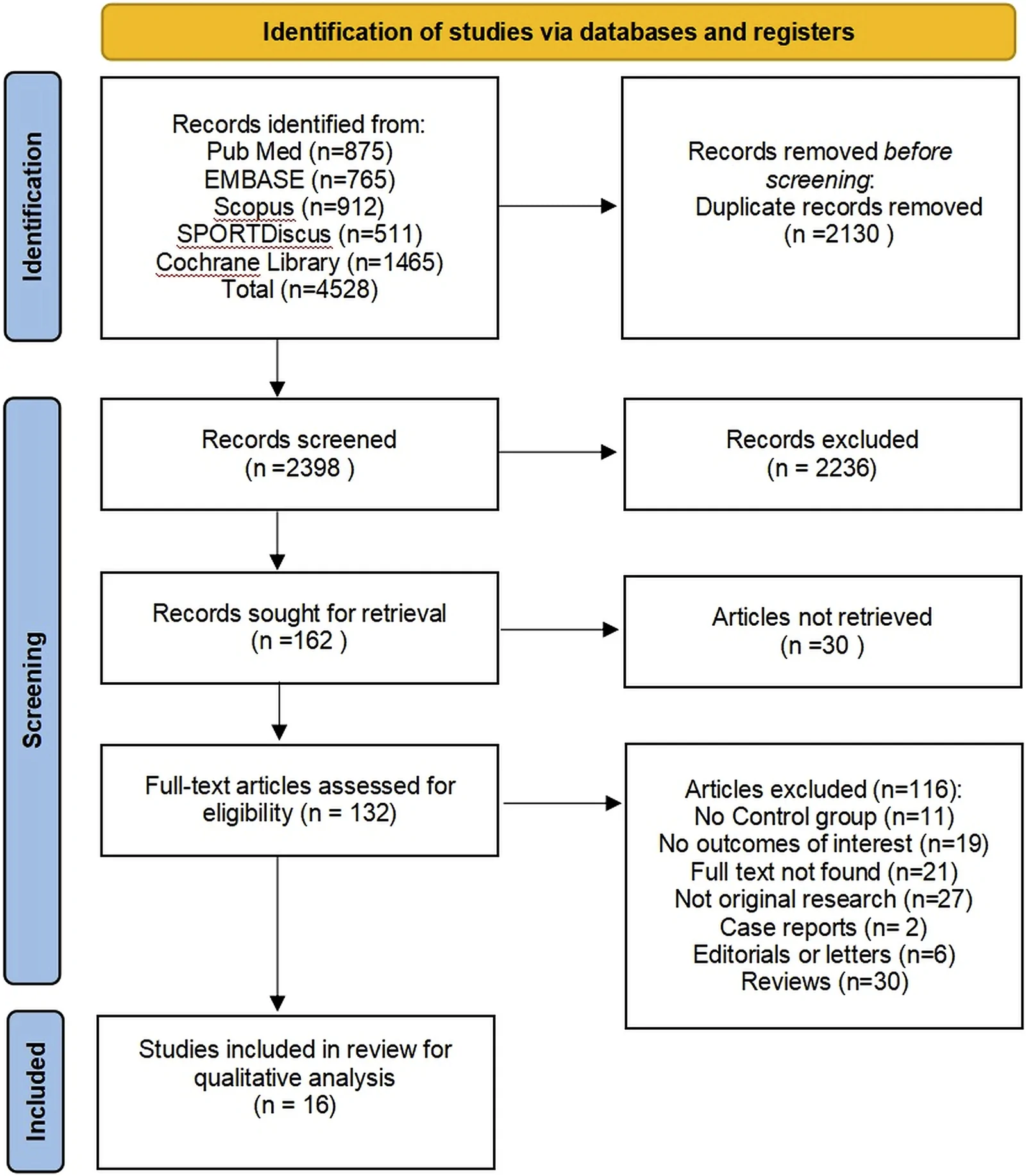
PRISMA 2020 flow diagram of study selection. A total of 4,528 records were identified, 2,398 screened, 132 assessed in full text, and 16 studies included.

During study selection and data extraction, minor disagreements occurred mainly regarding eligibility assessment and outcome classification. All disagreements were resolved through discussion between the reviewers, and no third reviewer adjudication was required. No study authors were contacted because the data required for the meta-analysis were available from the published articles and Supplementary Material.

### Study characteristics

The characteristics of the included studies are summarized in Table 1. A total of 550 animals were included across the 16 controlled animal studies, with 275 animals in the stem cell intervention groups and 275 animals in the control groups. The studies were published between 2007 and 2025 and were conducted in China, Japan, Korea, Singapore, and the Netherlands. The majority employed rabbit (n = 11) or rat (n = 5) ACL reconstruction models. Various stem cell types were used, including bone marrow-derived stem cells (BMSCs), adipose-derived stem cells (ADSCs), tendon-derived stem cells (TDSCs), synovium-derived stem cells (SMSCs), and human umbilical cord blood MSCs (HUCB-MSCs). Delivery strategies included direct intra-articular injection, graft wrapping, scaffold-based delivery, and bio-printed constructs. Follow-up times ranged from 2 to 24 weeks, and outcomes were assessed using biomechanical properties (ultimate failure load, stiffness), micro-CT analysis (bone volume fraction, BV/TV), and histological evaluation.

**TABLE 1:** Characteristics of the included articles.

Study	Region	Experimental animal	Stem cell type	Group size of each outcome at each timepoint	Control intervention	Experimental intervention	Follow-up timepoints	Outcome measures
(Xu et al., 2022)	China	rat	ADSC	EG n = 6; CG n = 6	ACLR + PBS intra-articular injection + hydrogel intratunnel injection; once intratunnel immediately after surgery + weekly intra-articular injection	ACLR + IPFP-MSC exosomes intra-articular injection + exosome-loaded hydrogel intratunnel injection; once intratunnel immediately after surgery + weekly intra-articular injection; exosome concentration 1 × 10^10^ particles/mL	4/8 weeks	Biomechanical Testing Micro-CT Scanning
(Teng et al., 2016)	China	rabbit	BMSC	EG n = 5; CG n = 5	ACLR alone	ACLR + BMSCs + PRP; BMSCs + PRP was locally applied to the graft/bone tunnel area once during surgery; total cell number 1 × 10^7^ BMSCs reported	8 weeks	Biomechanical Testing
(Tie et al., 2021)	China	rabbit	BMSC	EG n = 8; CG n = 8	ACLR + alginate gel	ACLR + BMSCs; BMSCs mixed with alginate gel were locally injected into the tendon–bone interface of tibial and femoral tunnels once during surgery; mean cell concentration: 1 × 10^7^ cells/mL, with 0.2 mL gel containing 2 × 10^6^ cells injected each time	12 weeks	Biomechanical Testing Micro-CT Scanning
(Li et al., 2022)	China	rat	BMSC	EG n = 6; CG n = 6	ACLR + PBS intra-articular injection; 50 μL PBS was injected into the joint cavity 3 times: immediately after surgery, postoperative day 3, and postoperative day 7	ACLR + BMSC-Exos; 50 μL BMSC-Exos was injected into the joint cavity 3 times: immediately after surgery, postoperative day 3, and postoperative day 7; exosome concentration: 1 × 10^11^ particles/mL	2/8 weeks	Biomechanical Testing Micro-CT Scanning
(Noh et al., 2024)	Korea	rat	SMSC	EG n = 7; CG n = 7	ACLR alone	ACLR + SMSC-derived scaffold-free fibrocartilage construct; the construct was locally applied to the tendon graft/bone tunnel interface once during surgery; this was a scaffold-free construct rather than injectable cell suspension	2/4 weeks	Biomechanical Testing
(Kosaka et al., 2016)	Japan	rabbit	ADSC	EG n = 8; CG n = 8	ACLR + fibrin glue only; the graft was coated with fibrin glue once during surgery	ACLR + ADRCs mixed with fibrin glue; ADRCs + fibrin glue were locally applied/coated onto the graft once during surgery; mean cell concentration: cells/mL not directly reported	2/4/6/8/12 weeks	Biomechanical Testing
(Matsumoto et al., 2021)	Japan	rabbit	ADSC	EG n = 7; CG n = 7	ACLR alone	ACLR + ADSC sheet; ADSC sheet was locally applied/transplanted around the semitendinosus tendon autograft once during surgery	2/4/8/16/24 weeks	Biomechanical Testing Micro-CT Scanning Histological Evaluation
(Soon et al., 2007)	Singapore	rabbit	BMSC	EG n = 7; CG n = 7	ACLR with Achilles tendon allograft alone	ACLR + autogenous MSCs in fibrin glue carrier; MSCs were coated onto the Achilles tendon allograft once before insertion into the bone tunnel during surgery; mean cell concentration: 3–4 × 10^6^ cells/mL	2/4/8 weeks	Biomechanical Testing
(Lui et al., 2014)	China	rat	TDSC	EG n = 5; CG n = 5	ACLR alone	ACLR + TDSC sheet; GFP-TDSC sheet was wrapped around the tendon graft once before graft insertion during surgery	2/6/12 weeks	Biomechanical Testing Micro-CT Scanning
(Tie et al., 2022)	China	rabbit	BMSC	EG n = 16; CG n = 16	ACLR + alginate gel	ACLR + BMSCs; BMSCs mixed with alginate gel were locally injected into the tendon–bone interface of tibial and femoral tunnels during surgery; injection number/time: intraoperative local injection, reported as 0.2 mL gel containing 2 × 10^6^ cells each time; mean cell concentration: 1 × 10^7^ cells/mL	4/12 weeks	Biomechanical Testing Micro-CT Scanning
(Yao et al., 2023)	China	rat	TDSC	EG n = 10; CG n = 10 (BIO) EG n = 6; CG n = 6 (CT)	ACLR alone	ACLR + decellularized TDSC sheet; dTDSC sheet was wrapped around the tendon graft once during ACLR	2/6 weeks	Biomechanical Testing Micro-CT Scanning
(Yanuar et al., 2025)	Netherlands	rabbit	ADSC	EG n = 4; CG n = 4	ACL primary repair alone	ACL primary repair + adipose-derived MSC-EVs; 100 μL EVs were injected once into the femoral tunnel at the bone–ligament interface during surgery; mean cell concentration: not reported/not applicable; EV dose: 40 μg/100 μL	6 weeks	Biomechanical Testing
(Dong et al., 2012)	China	rabbit	BMSC	EG n = 15; CG n = 15	ACLR + normal gastrocnemius tendon graft + fibrin glue coating	ACLR + BMSCs-Lv-Control; virus-infected BMSCs were immobilized in 0.2 mL fibrin glue and wrapped/coated onto the gastrocnemius tendon graft once immediately before insertion into the bone tunnels during surgery; mean cell concentration: not directly reported as cells/mL, but 1 × 10^7^ BMSCs in 0.2 mL fibrin glue was reported, calculated loading concentration ≈5 × 10^7^ cells/mL	4/8 weeks	Biomechanical Testing
(Lu et al., 2019)	China	rabbit	BMSC	EG n = 5; CG n = 5	ACLR + fresh-frozen allogenic semitendinous tendon graft + PBS bone-tunnel injection; 1 mL PBS was injected into the femoral and tibial bone tunnels once during surgery before graft implantation	ACLR + decellularized allogenic semitendinous tendon graft + autologous BMSCs; 1 mL BMSC suspension was seeded/deeply injected into the decellularized graft, and another 1 mL BMSC suspension was injected into the femoral and tibial bone tunnels once during surgery before graft implantation; mean cell concentration: 3.25 × 10^6^ cells/mL, approximately 6.5 × 10^6^ BMSCs used in total	2/4/8/12weeks	Biomechanical Testing Micro-CT Scanning
(Park et al., 2018)	Korea	rabbit	HUCB-MSC	EG n = 5; CG n = 5	ACLR + 3D bio-printed scaffold sleeve without MSCs	ACLR + 3D bio-printed scaffold sleeve seeded with MSCs; MSC-seeded scaffold sleeve was implanted into the bone tunnel once during surgery before graft passage	4/8/12weeks	Histological Evaluation
(Chen et al., 2023)	China	rabbit	ADSC	EG n = 6; CG n = 6	ACLR + saline local injection	ACLR + ADSCs + fibrin glue; ADSCs + fibrin glue was locally injected into the tendon–bone interface and tendon gap once during surgery; mean cell concentration: not reported	6/12 weeks	Biomechanical Testing

Abbreviations: ACLR, anterior cruciate ligament reconstruction; ACL, anterior cruciate ligament; ADSC, adipose-derived stem cell; ADRC, adipose-derived regenerative cell; BMSC, bone marrow-derived mesenchymal stem cell; De-BMSC, dedifferentiated osteogenic bone marrow-derived mesenchymal stem cell; SMSC, synovium-derived mesenchymal stem cell; TDSC, tendon-derived stem cell; dTDSC, decellularized tendon-derived stem cell sheet; HUCB-MSC, human umbilical cord blood-derived mesenchymal stem cell; IPFP-MSC, infrapatellar fat pad-derived mesenchymal stromal cell; MSC, mesenchymal stem/stromal cell; EV, extracellular vesicle; Exos, exosomes; PRP, platelet-rich plasma; PBS, phosphate-buffered saline; KGN, kartogenin; EG, experimental group; CG, control group; BIO, biomechanical testing; CT, micro-computed tomography; Micro-CT, micro-computed tomography.

### Risk of bias

The risk of bias assessment is presented in Table 2. Most studies were judged to have a low risk of bias in sequence generation and baseline comparability. However, domains such as allocation concealment, random housing, and blinding of caregivers were frequently rated as *unclear*. Only a few studies adequately reported blinding of outcome assessors. Selective outcome reporting and incomplete outcome data were generally considered at low risk. Overall, the methodological quality of the included animal studies was moderate, with the main limitations related to randomization and blinding.

**TABLE 2:** Results of the assessment of bias risk in animal studies.

Study	Sequence generation	Baseline characteristics	Allocation concealment	Random housing	Blinding of caregivers	Random outcome assessment	Blinding of outcome assessor	Incomplete outcome data	Selective outcome reporting	Other bias
Xu 2022	L	L	U	U	U	U	U	L	U	U
Teng 2016	L	L	U	U	U	U	L	L	U	U
Tie 2021	U	L	L	U	U	U	L	L	L	U
Li 2022	L	L	U	U	U	L	U	L	U	H
Noh 2024	L	L	U	U	U	U	U	L	U	U
Kosaka 2015	U	L	U	U	U	U	L	L	L	U
Matsumoto 2021	L	L	U	U	U	L	U	L	U	U
Michael 2007	L	L	L	U	L	U	U	L	U	U
Lui 2014	U	L	U	U	U	U	L	L	U	H
Tie 2022	L	L	U	U	U	U	U	L	U	U
Yao 2023	U	L	U	U	U	U	U	L	U	U
Yanuar 2025	U	L	U	U	L	L	L	L	U	U
Dong 2012	L	L	L	U	U	L	U	L	L	U
Lu 2019	U	L	U	U	U	L	U	L	U	U
Park 2017	L	L	U	U	U	L	L	L	U	H
Gang 2023	L	L	U	U	U	U	U	L	U	U

Abbreviations: L, denoting a low risk of bias, H indicating a high risk of bias, and U indicating an uncertain risk of bias.

### Meta-analysis outcomes

#### Ultimate failure load (UFL)

A total of 14 studies (n = 280 animals per group) were included in the pooled analysis. Stem cell therapy significantly enhanced the ultimate failure load compared to the control group (SMD = 1.34, 95% CI 0.93–1.75, P < 0.00001, I^2^ = 75%). Subgroup analysis by follow-up duration revealed consistent benefits at all time points (2, 4, 6, 8, and ≥12 weeks), with the most pronounced effect observed at 4 weeks (Figure 2).

**FIGURE 2:**
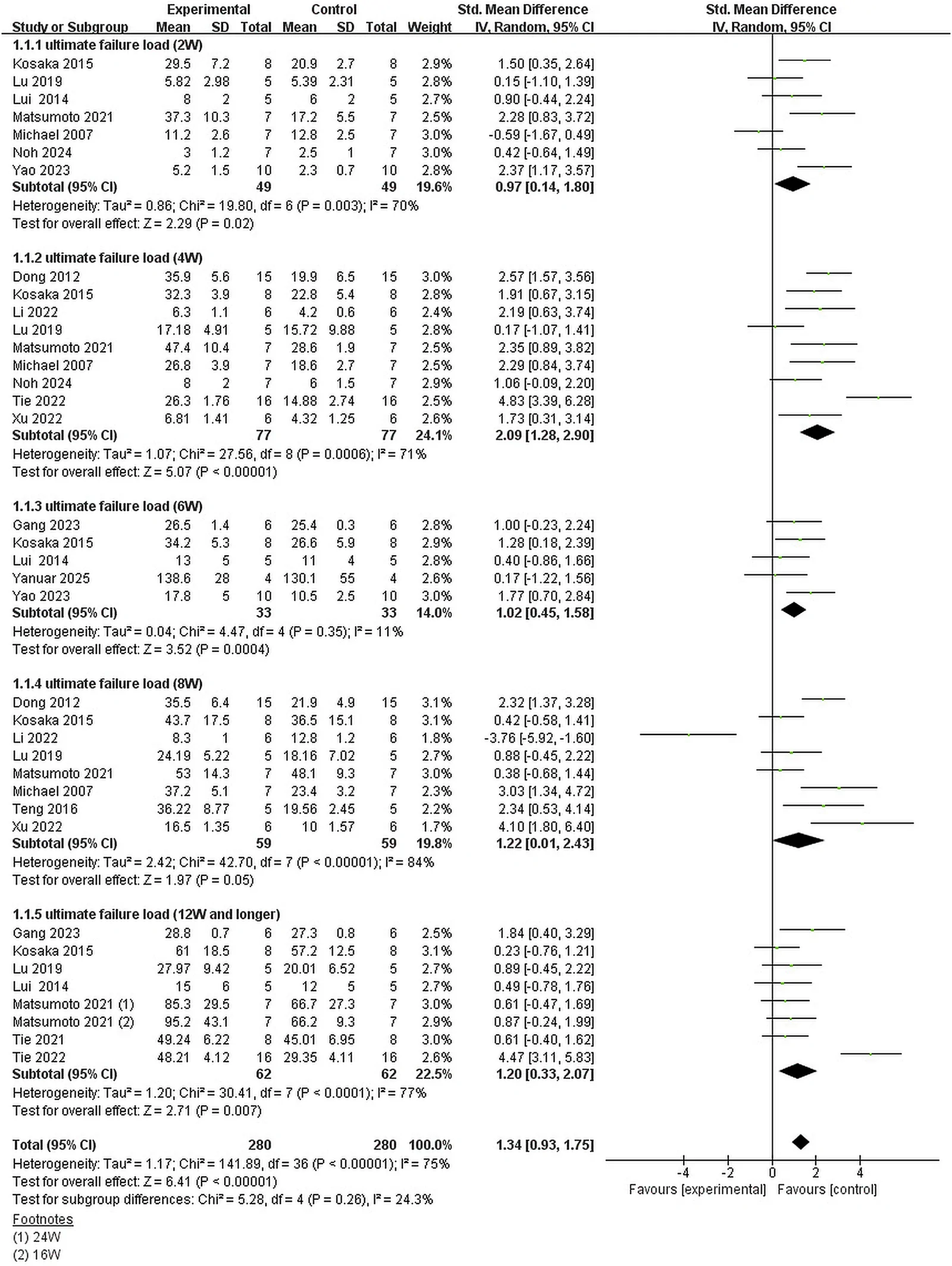
Ultimate Failure Load (UFL) Over Time with Stem Cell Interventions. A total of 14 studies (n = 280 animals per group) were included in the pooled analysis. Stem cell therapy significantly enhanced the ultimate failure load compared to the control group (SMD = 1.34, 95% CI 0.93–1.75, P < 0.00001, I^2^ = 75%). Subgroup analysis by follow-up duration revealed consistent benefits at all time points (2, 4, 6, 8, and ≥12 weeks), with the most pronounced effect observed at 4 weeks.

#### Stiffness

Thirteen studies (n = 264 animals per group) reported stiffness outcomes. The pooled results demonstrated a significant improvement in stiffness following stem cell therapy (SMD = 0.96, 95% CI 0.64–1.27, P < 0.00001, I^2^ = 61%). Subgroup analysis indicated no significant effect at 2 weeks (SMD = 0.60, 95% CI −0.31–1.50, P = 0.20, I^2^ = 75%), but substantial improvements were observed at later time points (4–12 weeks), suggesting a time-dependent effect (Figure 3).

**FIGURE 3:**
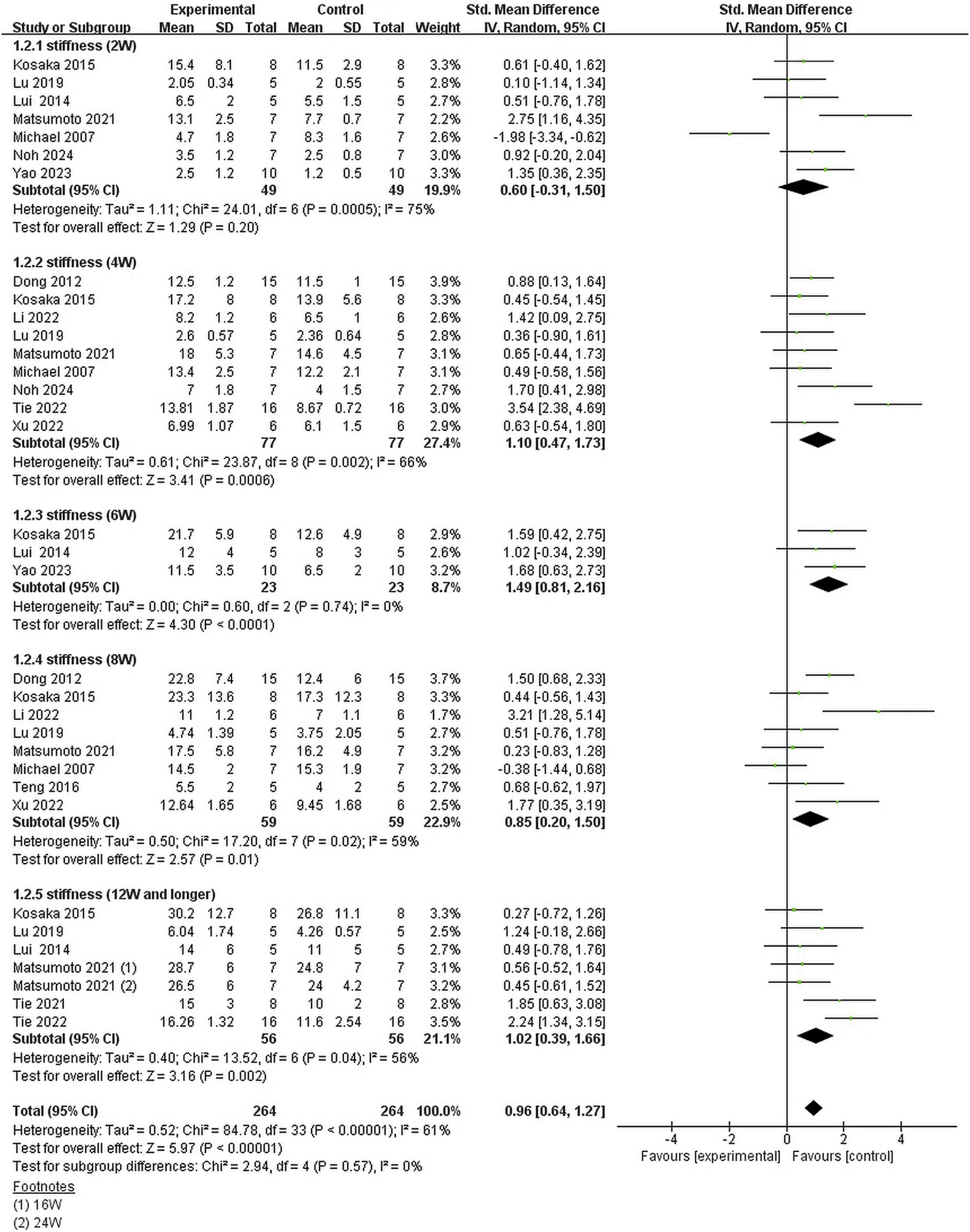
Stiffness Over Time with Stem Cell Interventions. Thirteen studies (n = 264 animals per group) reported stiffness outcomes. The pooled results demonstrated a significant improvement in stiffness following stem cell therapy (SMD = 0.96, 95% CI 0.64–1.27, P < 0.00001, I^2^ = 61%). Subgroup analysis indicated no significant effect at 2 weeks (SMD = 0.60, 95% CI -0.31–1.50, P = 0.20, I^2^ = 75%), but substantial improvements were observed at later time points (4–12 weeks), suggesting a time-dependent effect.

#### Bone volume fraction (BV/TV)

Seven studies (n = 144 animals per group) were included in the analysis of bone volume fraction. Stem cell interventions significantly increased bone volume fraction (SMD = 1.03, 95% CI 0.75–1.31, P < 0.00001, I^2^ = 76%). Subgroup analysis revealed that the effects were more pronounced at 4–6 weeks, aligning with the accelerated tendon-bone integration observed during the early healing phase (Figure 4).

**FIGURE 4:**
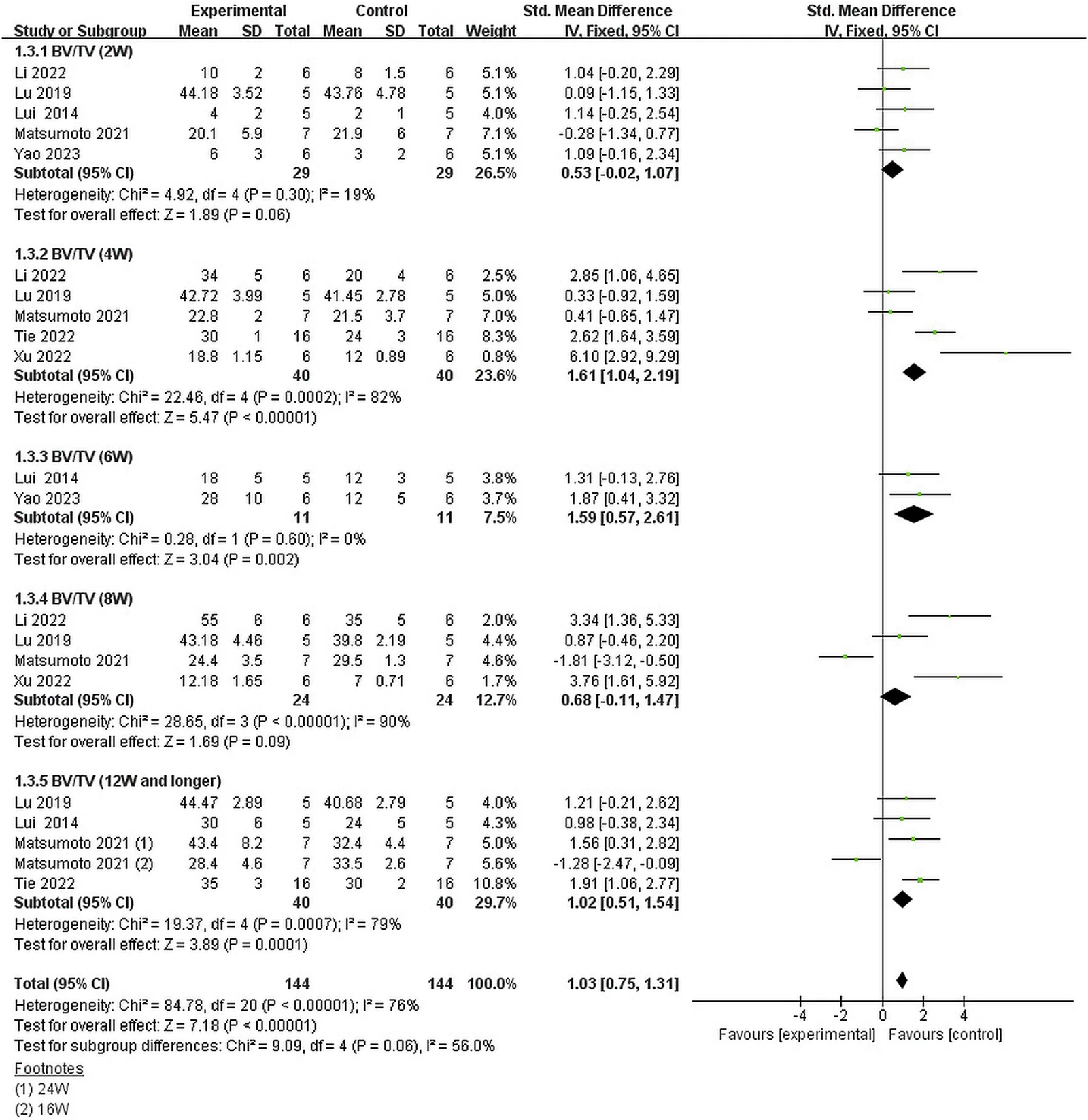
Bone Volume Fraction (BV/TV) Over Time with Stem Cell Interventions. Seven studies (n = 144 animals per group) were included in the analysis of bone volume fraction. Stem cell interventions significantly increased bone volume fraction (SMD = 1.03, 95% CI 0.75–1.31, P < 0.00001, I^2^ = 76%). Subgroup analysis revealed that the effects were more pronounced at 4–6 weeks, aligning with the accelerated tendon-bone integration observed during the early healing phase.

#### Histological evaluation

Two studies, comprising 36 animals in the stem cell intervention arms and 36 animals in the control arms, were included in the pooled analysis of histological evaluation. Stem cell-based interventions significantly improved histological scores for tendon-bone interface healing compared with controls (SMD = 0.86, 95% CI 0.59–1.13, P < 0.00001, I^2^ = 75%). The limited number of included studies should be considered when interpreting this result. Substantial heterogeneity was observed (I^2^ = 72%), which may be attributed to differences between the studies (Figure 5).

**FIGURE 5:**
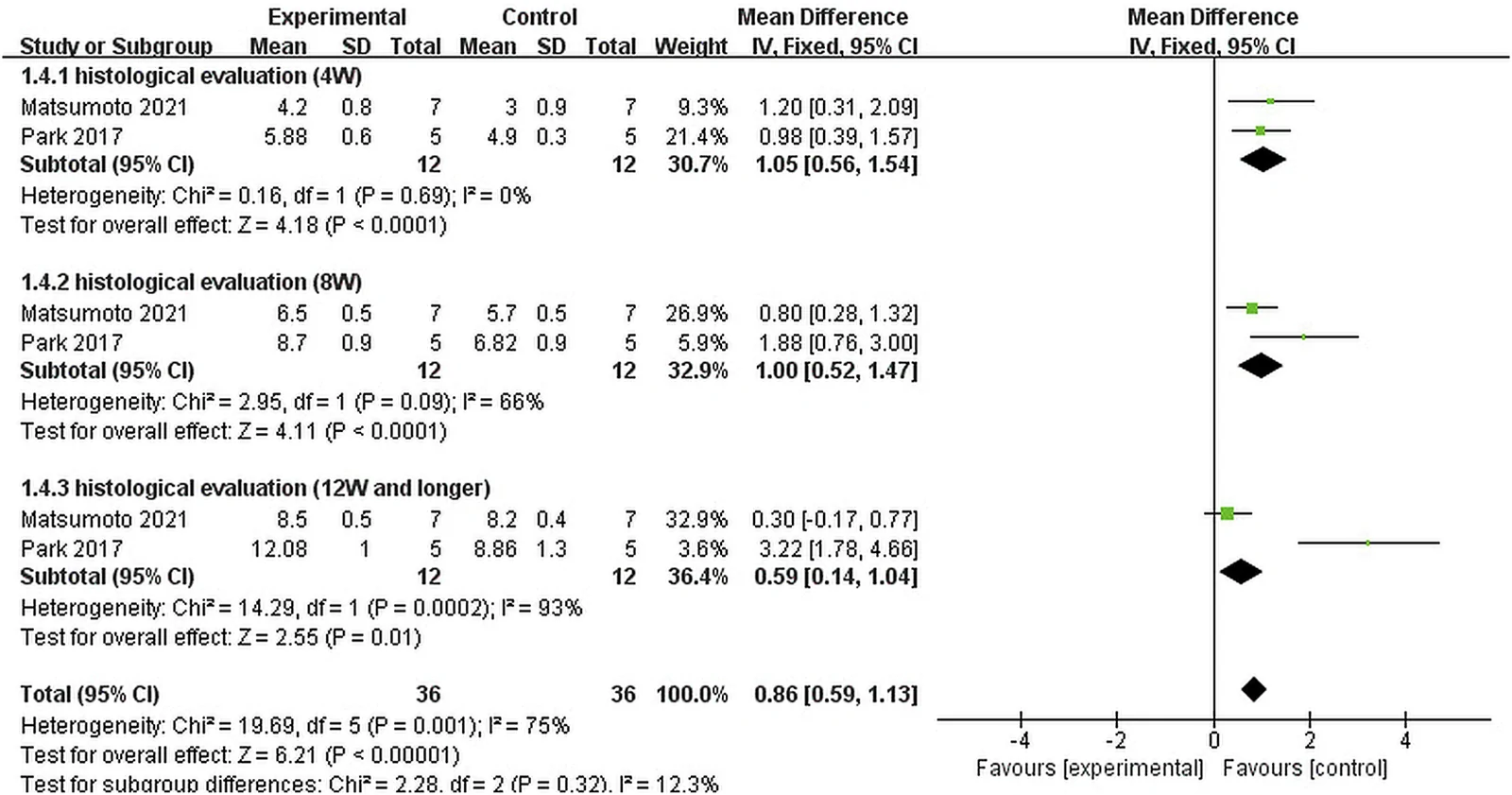
Histological Evaluation Over Time with Stem Cell Interventions. Two studies (n = 36 animals per group) were included in the histological evaluation. Stem cell interventions significantly improved the histological scores for tendon-bone interface healing compared to controls (SMD = 0.86, 95% CI 0.59–1.13, P < 0.00001, I^2^ = 75%). Substantial heterogeneity was observed (I^2^ = 72%), which may be attributed to differences between the studies.

### Subgroup analysis

In the subgroup analysis, the effects of stem cell therapies on the ultimate failure load (UFL), stiffness, bone volume fraction (BV/TV), and histological evaluation were assessed at various time points (2W, 4W, 6W, 8W, and 12W+). The following results were observed:

#### Ultimate failure load (UFL)

Stem cell interventions demonstrated the highest effect at 4 weeks (SMD = 2.09), with a significant drop at later time points. The overall trend showed a reduction in the effectiveness of stem cells with time, indicating a potential time-dependent effect on the UFL.

#### Stiffness

Significant improvements in stiffness were noted at 4–6 weeks (SMD = 1.49 at 6W), with a slight decline at 8W and 12W+ (SMD = 0.85 and 1.02, respectively). This suggests a peak response around 6 weeks, followed by a plateau or slight decrease.

#### Bone volume fraction (BV/TV)

The greatest increase in bone volume fraction was observed at 4W (SMD = 1.61), with a slight decrease at later time points, particularly at 8W (SMD = 0.68), indicating a rapid integration of the graft-bone interface in the early stages of healing.

#### Histological evaluation

The histological results showed a gradual decrease in SMD from 4W (SMD = 1.05) to 12W+ (SMD = 0.59), indicating diminishing improvements in tendon-bone interface healing over time.

These findings suggest that the effects of stem cell interventions vary over time, with the most significant improvements observed in the early stages (4–6 weeks) of healing. For a visual representation of these effects over time, please refer to Figure 6, which displays the changes in SMD for UFL, stiffness, BV/TV, and histological evaluation across the different time points.

**FIGURE 6:**
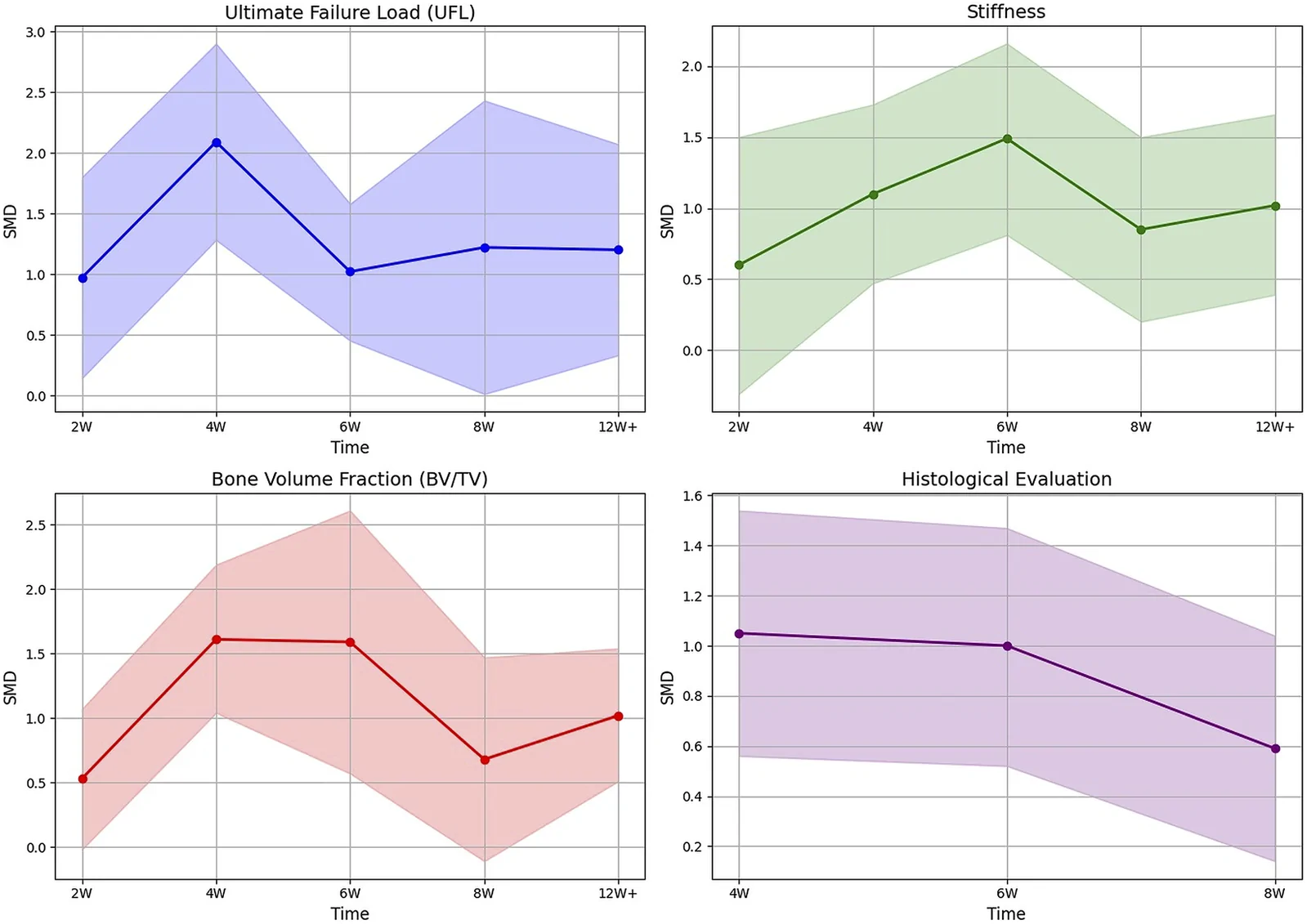
Changes in Ultimate Failure Load (UFL), Stiffness, Bone Volume Fraction (BV/TV), and Histological Evaluation Over Time with Stem Cell Interventions. This figure shows the standardized mean differences (SMD) for each outcome at different time points (2W, 4W, 6W, 8W, and 12W+), with shaded areas representing the 95% confidence intervals.

### Sensitivity analyses

Exploratory sensitivity analyses were performed to assess the robustness of the pooled estimates. All eligible studies were retained in the primary meta-analysis. The sensitivity analyses, presented in Supplementary File S3, were used only to explore the potential influence of individual studies on heterogeneity and did not alter the primary inclusion of studies or the main conclusions.

### Publication bias

Funnel plot inspection did not reveal obvious asymmetry for UFL, stiffness, or BV/TV. Egger’s regression test was not statistically significant (all P > 0.05), suggesting no strong evidence of publication bias. However, the relatively small number of studies per outcome limits the reliability of these assessments. The funnel plots in Supplementary Appendix S2 illustrate the distribution of studies for each outcome and further support our findings, indicating no apparent publication bias affecting the results.

## Discussion

This systematic review and meta-analysis synthesized evidence from 16 controlled animal studies to evaluate the effects of stem cell-based therapies on tendon-bone healing after ACL reconstruction. The results demonstrate that stem cell interventions significantly enhance biomechanical properties (ultimate failure load and stiffness) and bone volume fraction assessed by micro-CT compared with controls. Subgroup analyses further suggest that the beneficial effects are most pronounced at 4–6 weeks postoperatively, indicating a time-dependent role of stem cells in accelerating tendon-bone integration.

In this meta-analysis, the initial results showed high heterogeneity. The high heterogeneity could be attributed to several factors, including differences in animal models, stem cell sources, treatment methods, and follow-up durations. For instance, studies involving different animal species (e.g., rabbits and rats) may have significant biological differences that impact stem cell therapy outcomes. Additionally, the use of various stem cell types, such as bone marrow-derived mesenchymal stem cells (BMSCs), adipose-derived stem cells (ADSCs), and tendon-derived stem cells (TDSCs), as well as differing delivery methods (e.g., injection vs. scaffold), could contribute to inconsistent results. Furthermore, variations in follow-up periods could lead to different outcomes, especially considering that stem cell effects may diminish over time.

Substantial heterogeneity was observed across several outcomes. This may reflect clinical and methodological diversity among the included studies, including differences in animal species, graft types, stem cell sources, delivery strategies, outcome measurements, and follow-up durations. However, these explanations should be regarded as hypothesis-generating, because the limited number of studies precluded a reliable determination of the specific sources of heterogeneity. All eligible studies were retained in the primary meta-analysis, and exploratory sensitivity analyses are presented in Supplementary File S3.

### Comparison with previous literature

Our findings are broadly consistent with previous reviews and individual preclinical studies reporting potential biological benefits of stem cell-based strategies for graft incorporation. Xu et al. reported that mesenchymal stem cell-derived exosomes accelerated tendon-bone healing and intra-articular graft remodeling, while Park et al. demonstrated improved tendon-to-bone healing using three-dimensional bio-printed scaffold sleeves containing mesenchymal stem cells. Kosaka et al. reported that adipose-derived regenerative cells promoted tendon-bone healing in a rabbit model, and Yao et al. observed enhanced graft healing using bioactive decellularized tendon-derived stem cell sheets (Xu et al., 2022; Kosaka et al., 2016; Yao et al., 2023; Park et al., 2018). By quantitatively pooling the available animal data, our analysis adds quantitative support for the potential benefits of stem cell-based therapies in preclinical ACL reconstruction models.

However, the interpretation of these findings requires caution. The absence of consistent positive effects across all studies, outcomes, and time points should not be attributed solely to differences in cell source, dose, delivery method, animal species, or follow-up duration. It is also possible that stem cell-based interventions have limited, context-dependent, or non-reproducible effects under certain experimental conditions. In addition, the available literature may be affected by publication bias and selective reporting, because studies with negative or null results are less likely to be published. This concern is particularly relevant in the broader field of MSC-based therapies. Bezuglov et al. reported that, among 449 registered clinical trials of MSC injections in traumatology and orthopaedics, 159 trials had been completed and only 56 had published results in peer-reviewed journals, limiting the ability to form a complete judgment on efficacy and safety (Bezuglov et al., 2025). Therefore, although our pooled results suggest potential benefits of stem cell-based interventions in animal ACL reconstruction models, these findings should be interpreted as preliminary and should not be regarded as definitive evidence of efficacy.

### Potential mechanisms

The observed improvements likely reflect the multifaceted biological actions of stem cells. MSCs possess multipotent differentiation capacity, enabling them to generate fibrocartilage and bone-like tissue at the graft-bone interface. Moreover, their immunomodulatory effects may attenuate local inflammation, creating a permissive environment for tissue integration. Paracrine secretion of growth factors and extracellular vesicles can stimulate angiogenesis, enhance collagen fiber organization, and accelerate mineral deposition. Scaffold-assisted delivery and three-dimensional bio-printing approaches further optimize local cell retention and viability, contributing to superior tendon-bone healing (Higa et al., 2025; Song et al., 2023; Kim et al., 2025; Wang et al., 2025).

### Translational implications

Clinically, graft failure due to poor tendon-bone healing remains a major challenge in ACL reconstruction, with revision rates reported as high as 20%. The consistent preclinical evidence provided here underscores the translational potential of MSC-based strategies as biological adjuvants. If validated in well-designed clinical trials, stem cell-assisted ACL reconstruction could reduce tunnel widening, improve long-term graft durability, and ultimately enhance patient outcomes. Furthermore, stem cell-derived products such as exosomes may represent a safer, more standardized alternative to direct cell transplantation. From a translational perspective, the heterogeneity in dose reporting and administration timing limits the ability to define an optimal therapeutic regimen. Future preclinical studies should report the exact cell number or extracellular vesicle amount, delivery vehicle, anatomical site of administration, and timing relative to ACL reconstruction to facilitate reproducibility and clinical translation.

## Limitations

Several limitations should be acknowledged. First, heterogeneity across studies was substantial (I^2^ = 61%–76%), reflecting differences in animal species, graft types, stem cell sources, delivery strategies, outcome measurements, follow-up durations, and reporting quality. Although exploratory sensitivity analyses were conducted, the limited number of studies prevented reliable identification of the specific sources of heterogeneity.

Second, the methodological quality of the included animal studies was moderate. Key safeguards, including allocation concealment, random housing, random outcome assessment, and blinding, were incompletely reported in several studies, which may increase the risk of bias.

Third, the available evidence may be influenced by publication bias and selective reporting. Negative or null findings may be less likely to be published, potentially leading to overestimation of treatment effects. This issue is relevant to the broader MSC literature; Bezuglov et al. reported that among 449 registered clinical trials of MSC injections in traumatology and orthopaedics, only 56 had published results in peer-reviewed journals.

Finally, dosing and administration timing were inconsistently reported, and the included studies used different intervention formats, such as cell suspensions, scaffold-seeded cells, cell sheets, and extracellular vesicles. Therefore, dose-response analysis and identification of an optimal therapeutic window were not feasible. Because most studies used short-term animal models, the present findings should be interpreted as preliminary preclinical evidence rather than definitive proof of clinical efficacy.

## Future directions

Future preclinical studies should prioritize methodological rigor, including randomization, allocation concealment, and blinded outcome assessment, in accordance with ARRIVE guidelines. Comparative studies directly evaluating different stem cell sources, delivery routes, and scaffold systems are needed to identify the most effective strategies. Long-term experiments assessing graft durability, joint stability, and prevention of post-traumatic osteoarthritis will be essential to bridge the gap toward clinical translation. Moreover, mechanistic studies focusing on paracrine signaling, extracellular vesicles, and immunomodulatory pathways may provide insights into optimizing regenerative therapies.

## Conclusion

This systematic review and meta-analysis provides the first quantitative synthesis of animal studies evaluating stem cell therapies for tendon-bone healing after ACL reconstruction. Stem cell interventions significantly improve biomechanical, micro-CT outcomes and histological evaluation, with time-dependent benefits most evident during the early to mid phases of healing. While these findings highlight the promise of stem cell-based strategies, methodological heterogeneity and the limitations of preclinical models warrant cautious interpretation. Further standardized animal studies and well-designed clinical trials are needed to clarify the optimal approaches and confirm translational value.

## Glossary

3D: Three-dimensional
ACL: Anterior cruciate ligament
ACLR: Anterior cruciate ligament reconstruction
ADRCs: Adipose-derived regenerative cells
ADSCs: Adipose-derived stem cells
ARRIVE: Animal Research: Reporting of *In Vivo* Experiments
BIO: Biomechanical testing
BMSCs: Bone marrow-derived mesenchymal stem cells
BV/TV: Bone volume fraction/Tissue volume
CG: Control group
CI: Confidence interval
CT: Computed tomography
De-BMSCs: Dedifferentiated osteogenic bone marrow-derived mesenchymal stem cells
dTDSC: Decellularized tendon-derived stem cell
EG: Experimental group
EVs: Extracellular vesicles
HUCB-MSCs: Human umbilical cord blood-derived mesenchymal stem cells
**I** ^**2**^: Inconsistency index
IPFP-MSCs: Infrapatellar fat pad-derived mesenchymal stromal cells
KGN: Kartogenin
MD: Mean difference
MeSH: Medical Subject Headings
Micro-CT: Micro-computed tomography
MSCs: Mesenchymal stem/stromal cells
PBS: Phosphate-buffered saline
PRISMA: Preferred Reporting Items for Systematic Reviews and Meta-Analyses
PROSPERO: International Prospective Register of Systematic Reviews
PRP: Platelet-rich plasma
SMD: Standardized mean difference
SMSCs: Synovium-derived mesenchymal stem cells
SYRCLE: Systematic Review Centre for Laboratory Animal Experimentation
TDSCs: Tendon-derived stem cells
UFL: Ultimate failure load

## References

[B1] Bezuglov E. Dolgalev I. Kuznetsova M. Zholinskiy A. Savin E. Goncharov E. (2025). Mesenchymal stem cells injections in traumatology and orthopaedics: common practice or still a promising area with many uncertainties? BMC Musculoskelet. Disord. 26 (1), 840. 10.1186/s12891-025-09123-8 40898126 PMC12403583

[B2] Chen G. Zheng Q. Liu M. He H. Ju X. Jiang K. (2023). Effect of kartogenin combined with adipose-derived stem cells on tendon-bone healing after anterior cruciate ligament reconstruction. Zhongguo Xiu Fu Chong Jian Wai Ke Za Zhi 37 (8), 1002–1010. Chinese. 10.7507/1002-1892.202305011 37586802 PMC10435343

[B3] Cho W. S. Chung S. G. Kim W. Jo C. H. Lee S. U. Lee S. Y. (2021). Mesenchymal stem cells use in the treatment of tendon disorders: a systematic review and meta-analysis of prospective clinical studies. Ann. Rehabil. Med. 45 (4), 274–283. 10.5535/arm.21078 34496470 PMC8435464

[B4] Dong Y. Zhang Q. Li Y. Jiang J. Chen S. (2012). Enhancement of tendon-bone healing for anterior cruciate ligament (ACL) reconstruction using bone marrow-derived mesenchymal stem cells infected with BMP-2. Int. J. Mol. Sci. 13 (10), 13605–13620. 10.3390/ijms131013605 23202970 PMC3497344

[B5] Guo R. Chen M. Chen Y. Chen R. Liu H. Chen L. (2018). Current evidence of adult stem cells to enhance anterior cruciate ligament treatment: a systematic review of animal trials. Arthroscopy 34 (1), 331–340. 10.1016/j.arthro.2017.07.010 28967542

[B6] Higa K. Murata D. Azuma C. Nishida K. Nakayama K. (2025). Promotion of bone-tendon healing after ACL reconstruction using scaffold-free constructs comprising ADSCs produced by a bio-3D printer in rabbit models. J. Orthop. Transl. 52, 265–275. 10.1016/j.jot.2025.03.019 40342550 PMC12059222

[B7] Huang Y. He B. Wang L. Yuan B. Shu H. Zhang F. (2020). Bone marrow mesenchymal stem cell-derived exosomes promote rotator cuff tendon-bone healing by promoting angiogenesis and regulating M1 macrophages in rats. Stem Cell Res. Ther. 11 (1), 496. 10.1186/s13287-020-02005-x 33239091 PMC7687785

[B8] Kim W. J. Kwon D. R. Lee H. Lee J. Y. Moon Y. S. Lee S. C. (2025). 3D bioprinted multi-layered cell constructs with gradient core-shell interface for tendon-to-bone tissue regeneration. Bioact. Mater 43, 471–490. 10.1016/j.bioactmat.2024.10.002 40115882 PMC11923439

[B9] Kosaka M. Nakase J. Hayashi K. Tsuchiya H. (2016). Adipose-derived regenerative cells promote tendon-bone healing in a rabbit model. Arthroscopy 32 (5), 851–859. 10.1016/j.arthro.2015.10.012 26790583

[B10] Li Z. Li Q. Tong K. Zhu J. Wang H. Chen B. (2022). BMSC-derived exosomes promote tendon-bone healing after anterior cruciate ligament reconstruction by regulating M1/M2 macrophage polarization in rats. Stem Cell Res. Ther. 13 (1), 295. 10.1186/s13287-022-02975-0 35841008 PMC9284827

[B11] Lu W. Xu J. Dong S. Xie G. Yang S. Huangfu X. (2019). Anterior cruciate ligament reconstruction in a rabbit model using a decellularized allogenic semitendinous tendon combined with autologous bone marrow-derived mesenchymal stem cells. Stem Cells Transl. Med. 8 (9), 971–982. 10.1002/sctm.18-0132 31077578 PMC6708071

[B12] Lui P. P. Wong O. T. Lee Y. W. (2014). Application of tendon-derived stem cell sheet for the promotion of graft healing in anterior cruciate ligament reconstruction. Am. J. Sports Med. 42 (3), 681–689. 10.1177/0363546513517539 24451112

[B13] Matsumoto T. Sato Y. Kobayashi T. Suzuki K. Kimura A. Soma T. (2021). Adipose-derived stem cell sheets improve early biomechanical graft strength in rabbits after anterior cruciate ligament reconstruction. Am. J. Sports Med. 49 (13), 3508–3518. 10.1177/03635465211041582 34643475

[B14] Mayer M. A. Deliso M. Hong I. S. Saltzman B. M. Longobardi R. S. DeLuca P. F. (2025). Rehabilitation and return to play protocols after anterior cruciate ligament reconstruction in soccer players: a systematic review. Am. J. Sports Med. 53 (1), 217–227. 10.1177/03635465241233161 38622858

[B15] Noh S. Lee S. J. Yoo J. J. Jin Y. J. Yun H. W. Min B. H. (2024). Synovium-derived mesenchymal stem cell-based scaffold-free fibrocartilage engineering for bone-tendon interface healing in an anterior cruciate ligament reconstruction model. Tissue Eng. Regen. Med. 21 (2), 341–351. 10.1007/s13770-023-00593-2 37856071 PMC10825091

[B16] Park S. H. Choi Y. J. Moon S. W. Lee B. H. Shim J. H. Cho D. W. (2018). Three-dimensional bio-printed scaffold sleeves with mesenchymal stem cells for enhancement of tendon-to-bone healing in anterior cruciate ligament reconstruction using soft-tissue tendon graft. Arthroscopy 34 (1), 166–179. 10.1016/j.arthro.2017.04.016 28688825

[B17] Rothrauff B. B. Jorge A. de Sa D. Kay J. Fu F. H. Musahl V. (2020). Anatomic ACL reconstruction reduces risk of post-traumatic osteoarthritis: a systematic review with minimum 10-year follow-up. Knee Surg. Sports Traumatol. Arthrosc. 28 (4), 1072–1084. 10.1007/s00167-019-05665-2 31471726

[B18] Song L. Li N. Chen J. (2025). Research advances in mesenchymal stem cells and related therapies for rotator cuff tendon-to-bone healing. Front. Bioeng. Biotechnol. 13, 1647909. 10.3389/fbioe.2025.1647909 40901255 PMC12399614

[B19] Song W. Zhang D. Wu D. Zhong L. Chen H. Li Q. (2023). Cryopreserved adipose-derived stem cell sheets as an off-the-shelf scaffold for augmenting tendon-to-bone healing in a rabbit model of chronic rotator cuff tear. Am. J. Sports Med. 51 (8), 2005–2017. 10.1177/03635465231171682 37227145

[B20] Soon M. Y. Hassan A. Hui J. H. Goh J. C. Lee E. H. (2007). An analysis of soft tissue allograft anterior cruciate ligament reconstruction in a rabbit model: a short-term study of the use of mesenchymal stem cells to enhance tendon osteointegration. Am. J. Sports Med. 35 (6), 962–971. 10.1177/0363546507300057 17400750

[B21] Teng C. Zhou C. Xu D. Bi F. (2016). Combination of platelet-rich plasma and bone marrow mesenchymal stem cells enhances tendon-bone healing in a rabbit model of anterior cruciate ligament reconstruction. J. Orthop. Surg. Res. 11 (1), 96. 10.1186/s13018-016-0433-7 27605093 PMC5015347

[B22] Tie K. Cai J. Qin J. Xiao H. Shangguan Y. Wang H. (2021). Nanog/NFATc1/Osterix signaling pathway-mediated promotion of bone formation at the tendon-bone interface after ACL reconstruction with De-BMSCs transplantation. Stem Cell Res. Ther. 12 (1), 576. 10.1186/s13287-021-02643-9 34775995 PMC8591902

[B23] Tie K. Cai J. Shi H. Li X. Shangguan Y. Chen L. (2022). Autologous dedifferentiated osteogenic bone marrow mesenchymal stem cells promote bone formation in a rabbit model of anterior cruciate ligament reconstruction *versus* bone marrow mesenchymal stem cells. Arthroscopy 38 (7), 2246–2254.e1. 10.1016/j.arthro.2022.01.021 35093495

[B24] van Melick N. Senorski E. H. Królikowska A. Prill R. (2025). Anterior cruciate ligament reconstruction rehabilitation: decades of change. Knee Surg. Sports Traumatol. Arthrosc. 33 (4), 1178–1182. 10.1002/ksa.12600 39925169

[B25] Wang H. D. Li Z. Hu X. Ao Y. (2022). Efficacy of stem cell therapy for tendon graft ligamentization after anterior cruciate ligament reconstruction: a systematic review. Orthop. J. Sports Med. 10 (6), 23259671221098363. 10.1177/23259671221098363 35706553 PMC9189545

[B26] Wang D. R. Xu Y. Wang J. Chen H. Li J. Wu J. (2023). Extracellular vesicles: emerged as a promising strategy for tendon regeneration. World J. Stem Cells 15 (4), 165–182. 10.4252/wjsc.v15.i4.165 37181006 PMC10173817

[B27] Wang J. Li Q. Li Z. Song X. Wang Y. Yang H. (2024). Mesenchymal stem cell-derived extracellular vesicles accelerate tendon-bone healing by delivering BMP-2, promoting bone and fibrocartilage formation *via* the Smad/RUNX2 pathway. Cell Death Discov. 10 (1), 334. 10.1038/s41420-024-01973-w 39043648

[B28] Wang Y. Chen C. Zhang C. Cheng J. An B. Zhang N. (2025). 3D bioprinting for tendon-bone interface regeneration. Int. J. Bioprint 11 (2), 164–183. 10.36922/ijb.8411

[B29] Xu J. Ye Z. Han K. Zheng T. Zhang T. Dong S. (2022). Infrapatellar fat pad mesenchymal stromal cell-derived exosomes accelerate tendon-bone healing and intra-articular graft remodeling after anterior cruciate ligament reconstruction. Am. J. Sports Med. 50 (3), 662–673. 10.1177/03635465211072227 35224997

[B30] Yang C. Teng Y. Geng B. Xiao H. Chen C. Chen R. (2023). Strategies for promoting tendon-bone healing: current status and prospects. Front. Bioeng. Biotechnol. 11, 1118468. 10.3389/fbioe.2023.1118468 36777256 PMC9911882

[B31] Yanuar A. Agustina H. Antarianto R. D. Hidajat N. N. Mahyuddin A. I. Dilogo I. H. (2025). Extracellular vesicles from adipose-derived mesenchymal stem cells improve ligament-bone integration after anterior cruciate ligament primary repair in rabbit. Biomolecules 15 (3), 396. 10.3390/biom15030396 40149932 PMC11940348

[B32] Yao S. Liang Z. Lee Y. W. Yung P. S. H. Lui P. P. Y. (2023). Bioactive decellularized tendon-derived stem cell sheet for promoting graft healing after anterior cruciate ligament reconstruction. Am. J. Sports Med. 51 (1), 66–80. 10.1177/03635465221135770 36592017

[B33] Yuan C. Song W. Jiang X. Wang Y. Li C. Yu W. (2024). Adipose-derived stem cell-based optimization strategies for musculoskeletal regeneration: recent advances and perspectives. Stem Cell Res. Ther. 15, 91. 10.1186/s13287-024-03703-6 38539224 PMC10976686

[B34] Zhang X. Song W. Liu Y. Han K. Wu Y. Cho E. (2024). Healthy tendon stem cell-derived exosomes promote tendon-to-bone healing of aged chronic rotator cuff tears by breaking the positive-feedback cross-talk between senescent tendon stem cells and macrophages through modulation of macrophage polarization. Small 20 (31), e2311033. 10.1002/smll.202311033 38459643

