# Supplementary material for: Effect of stem cell therapies on tendon-bone healing after anterior cruciate ligament reconstruction in animal models: a systematic review and meta-analysis
Source: Front Bioeng Biotechnol. 2026 Oct 20;14:1869863. doi: 10.3389/fbioe.2026.1869863 (PMC13728609; doi:10.3389/fbioe.2026.1869863)
Supplement: Supplementary file 1 [file Table1.docx]

Search strategies for the systematic review and meta-analysis on stem cell therapies for tendon–bone healing after anterior cruciate ligament (ACL) reconstruction in animal models. The following databases were searched from inception to August 2025.

| Database | Search strategy |
| --- | --- |
| PubMed | ("anterior cruciate ligament"[Mesh] OR "ACL"[tiab] OR "anterior cruciate ligament"[tiab]) AND ("reconstruction"[tiab] OR "reconstructive surgical procedures"[Mesh] OR "surgery"[tiab] OR "graft"[tiab]) AND ("stem cells"[Mesh] OR "stem cell"[tiab] OR "mesenchymal stem cell"[tiab] OR "MSC"[tiab] OR "bone marrow stem cell"[tiab] OR "adipose-derived stem cell"[tiab] OR "tendon-derived stem cell"[tiab] OR "synovium-derived stem cell"[tiab] OR "umbilical cord blood stem cell"[tiab] OR "exosomes"[tiab] OR "extracellular vesicles"[tiab] OR "tissue scaffold"[Mesh] OR "scaffold"[tiab]) AND ("animals"[Mesh] OR "animal experiment*"[tiab] OR "preclinical"[tiab] OR "in vivo"[tiab] OR "rat"[tiab] OR "rabbit"[tiab]) |
| Embase | ('anterior cruciate ligament'/exp OR 'anterior cruciate ligament':ab,ti OR ACL:ab,ti) AND ('reconstruction'/exp OR 'surgery'/exp OR reconstruction:ab,ti OR surgery:ab,ti OR graft:ab,ti) AND ('stem cell'/exp OR 'mesenchymal stem cell'/exp OR 'bone marrow stem cell'/exp OR 'adipose derived stem cell'/exp OR 'tendon derived stem cell'/exp OR 'synovium derived stem cell'/exp OR 'umbilical cord blood stem cell'/exp OR stem cell:ab,ti OR MSC:ab,ti OR exosome:ab,ti OR 'extracellular vesicle':ab,ti OR scaffold:ab,ti) AND ('animal experiment'/exp OR 'preclinical study'/exp OR 'in vivo study'/exp OR animal:ab,ti OR rat:ab,ti OR rabbit:ab,ti) |
| Scopus | TITLE-ABS-KEY ( "anterior cruciate ligament" OR ACL ) AND TITLE-ABS-KEY ( reconstruction OR surgery OR graft ) AND TITLE-ABS-KEY ( "stem cell" OR "mesenchymal stem cell" OR MSC OR "bone marrow stem cell" OR "adipose-derived stem cell" OR "tendon-derived stem cell" OR "synovium-derived stem cell" OR "umbilical cord blood stem cell" OR exosome OR "extracellular vesicle" OR scaffold ) AND TITLE-ABS-KEY ( animal OR rat OR rabbit OR preclinical OR "in vivo" ) |
| SPORTDiscus | ( "anterior cruciate ligament" OR ACL ) AND ( reconstruction OR surgery OR graft ) AND ( "stem cell" OR "mesenchymal stem cell" OR MSC OR "bone marrow stem cell" OR "adipose-derived stem cell" OR "tendon-derived stem cell" OR "synovium-derived stem cell" OR "umbilical cord blood stem cell" OR exosome OR "extracellular vesicle" OR scaffold ) AND ( animal OR rat OR rabbit OR preclinical OR "in vivo" ) |
| Cochrane Library | ("anterior cruciate ligament" OR ACL) AND (reconstruction OR surgery OR graft) AND ("stem cell" OR "mesenchymal stem cell" OR MSC OR "bone marrow stem cell" OR "adipose-derived stem cell" OR "tendon-derived stem cell" OR "synovium-derived stem cell" OR "umbilical cord blood stem cell" OR exosome OR "extracellular vesicle" OR scaffold) AND (animal OR rat OR rabbit OR preclinical OR "in vivo") |
