# Supplementary material for: Effect of stem cell therapies on tendon-bone healing after anterior cruciate ligament reconstruction in animal models: a systematic review and meta-analysis
Source: Front Bioeng Biotechnol. 2026 Oct 20;14:1869863. doi: 10.3389/fbioe.2026.1869863 (PMC13728609; doi:10.3389/fbioe.2026.1869863)
Supplement: Supplementary file 2 [file Table2.docx]

Funnel plot for

BV/TV

histological assessment

stiffness

ultimate failure load
