# Supplementary material for: Effect of stem cell therapies on tendon-bone healing after anterior cruciate ligament reconstruction in animal models: a systematic review and meta-analysis
Source: Front Bioeng Biotechnol. 2026 Oct 20;14:1869863. doi: 10.3389/fbioe.2026.1869863 (PMC13728609; doi:10.3389/fbioe.2026.1869863)
Supplement: Supplementary file 3 [file Table3.docx]

**Forest plot of Stiffness after excluding certain studies**

This forest plot presents the analysis of stiffness after excluding certain studies. Following the exclusion, the consistency of results across different time points (2 weeks, 4 weeks, 6 weeks, etc.) improved, and heterogeneity decreased. The effect of stem cell therapy on stiffness became more evident. The overall effect size (SMD = 0.92, 95% CI [0.69, 1.14]) suggests that stem cell therapy has a significant effect on improving stiffness.

**Forest plot of Ultimate Failure Load (UFL) after excluding certain studies**

This forest plot shows the analysis of Ultimate Failure Load (UFL) after excluding certain studies. After the exclusion, the effect of stem cell therapy on UFL was further validated across different time points, with a significant reduction in heterogeneity. The overall effect size (SMD = 1.21, 95% CI [0.86, 1.55]) indicates that stem cell therapy significantly enhances the ultimate failure load.

**Forest plot of Bone Volume Fraction (BV/TV) after excluding certain studies**

This forest plot shows the analysis of Bone Volume Fraction (BV/TV) after excluding certain studies. After the exclusion, the results became more consistent, with a significant reduction in heterogeneity (I²), further supporting the positive role of stem cell therapy in tendon–bone healing. The overall effect size (SMD = 1.32, 95% CI [1.02, 1.62]) indicates that stem cell therapy significantly improves bone volume fraction.
